# Supplementary material for: Dietary Intake of a Milk Sphingolipid-Rich MFGM/EV Concentrate Ameliorates Age-Related Metabolic Dysfunction
Source: Nutrients. 2025 Jul 31;17(15):2529. doi: 10.3390/nu17152529 (PMC12348648; doi:10.3390/nu17152529)
Supplement: Supplementary file 1 [file nutrients-17-02529-s001.zip › Figures S1-S5.pdf]

SUPPLEMENTAL INFORMATION

Figure S1

A Total number of lipids significantly different lipids

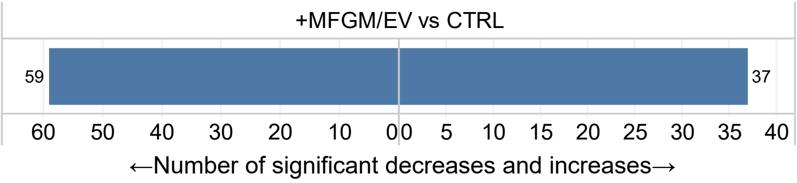

B Number of lipids significantly different lipids per class

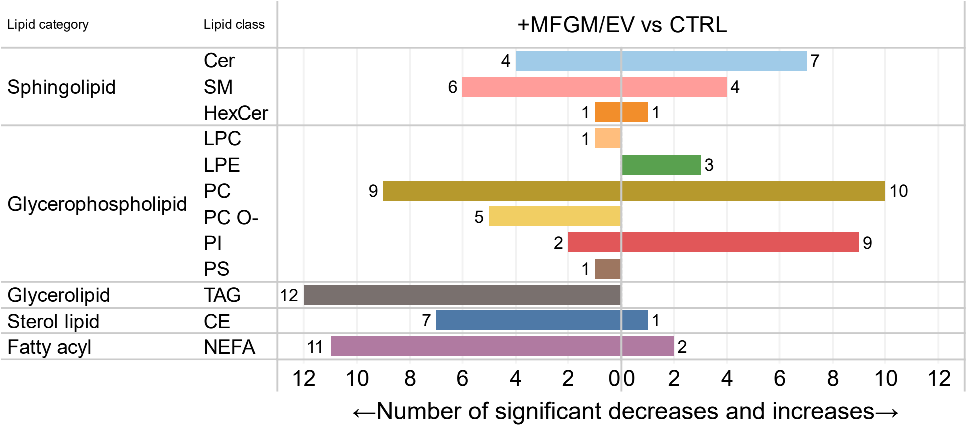

Figure S1. Frequencies of significantly different lipids in plasma. Related to Figure 2.

A) Total number of lipids with different plasma concentrations. Statistical analysis is done using ANOVA with multiple hypothesis correction. Lipids with q-value < 0.01 are counted as significantly different.

B) Number of lipids with different plasma concentrations per lipid class.

Figure S2

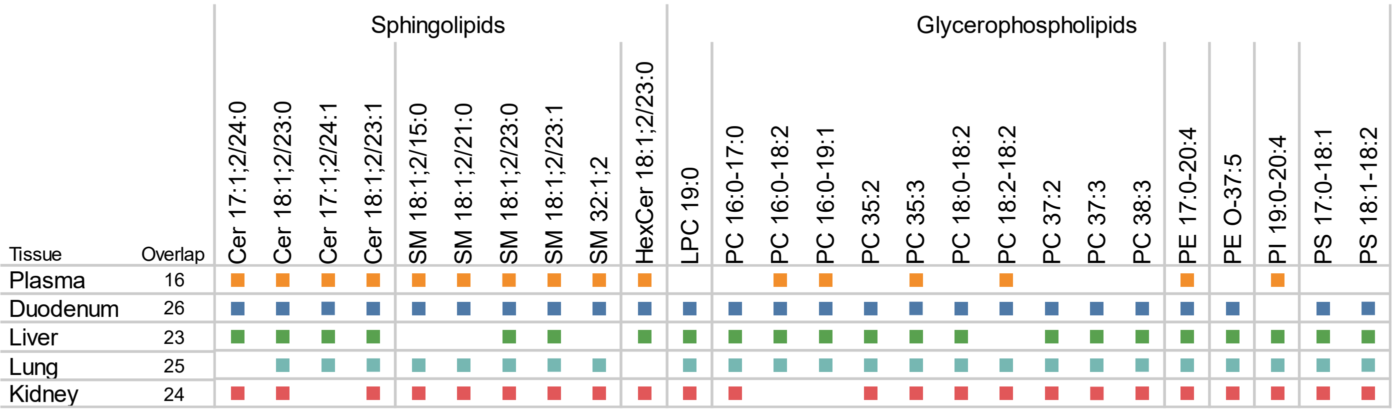

Figure S2. The MFGM/EV ingredient promotes accretion of very long odd-chain sphingolipids at the whole-body level. Related to Figure 3.

Overlap between significantly and consistently elevated lipids in plasma and tissues of elderly rats fed the MFGM/EV ingredient. Lipids with q-value < 0.05 and fold difference > 1 are considered as significantly different.

Figure S3

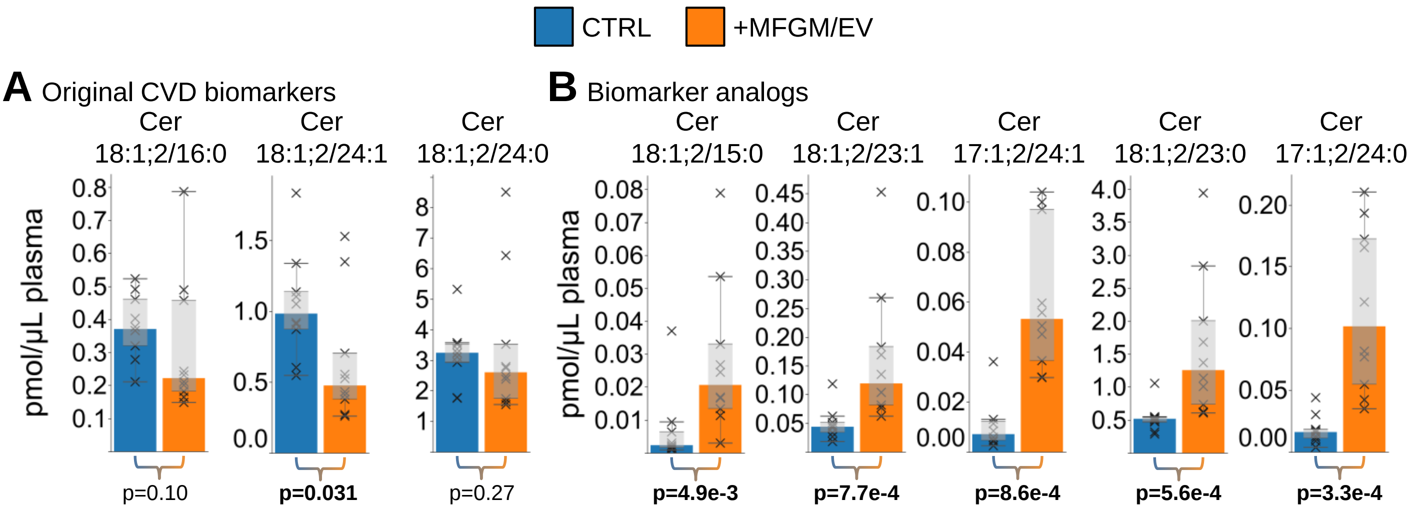

Figure S3. Plasma levels of ceramides. Related to Figure 5.

A) Concentrations of plasma ceramide biomarkers associated with a fatal outcome for patients with cardiovascular disease.

B) Plasma concentrations of ceramides that are structurally reminiscent of the even ceramide biomarkers and significantly increased by the MGFM/EV ingredient.

Data represent medians, box plots and crosses show individual values (n = 7-10 rats/group). Statistical analysis is done by non-parametric unpaired two-samples Wilcoxon test. Significant differences (p<0.05) are highlighted in bold.

Figure S4

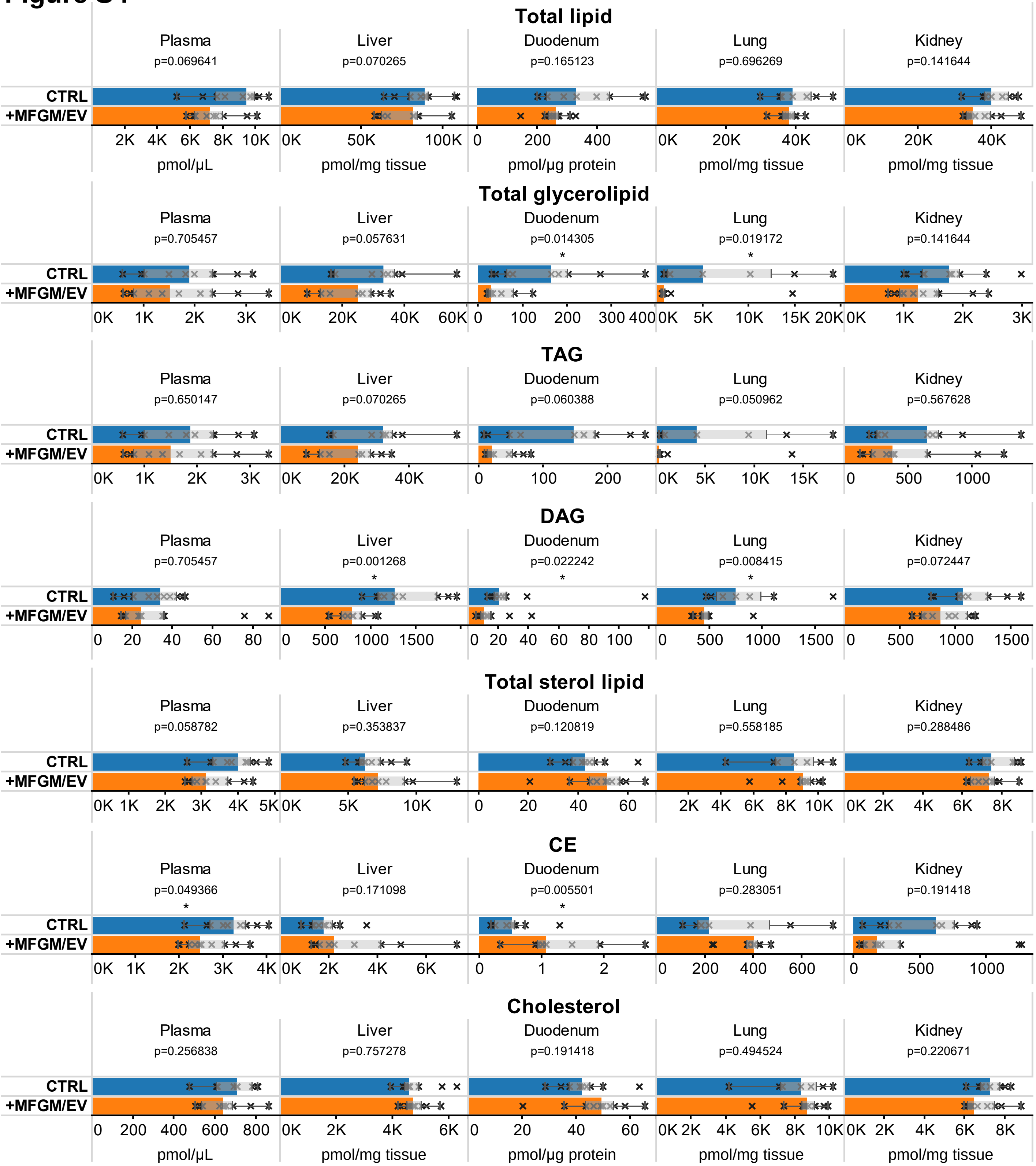

Figure S4. Lipid-lowering effects by the MFGM/EV ingredient. Related to Figure 5.

Total levels of indicated lipid categories and lipid classes in blood plasma, liver, duodenum, lung and kidney. Some plots are also shown in Fig. 5. Nonetheless, for comparative purposes all plots are shown together here. Data represent box plots, with bars indicating medians and crosses showing individual values (n=7-10 rats/condition). Statistical analysis is done by non-parametric Wilcoxon testing. Significant differences ($p < 0.05$) are highlighted by an asterisk.

Figure S5

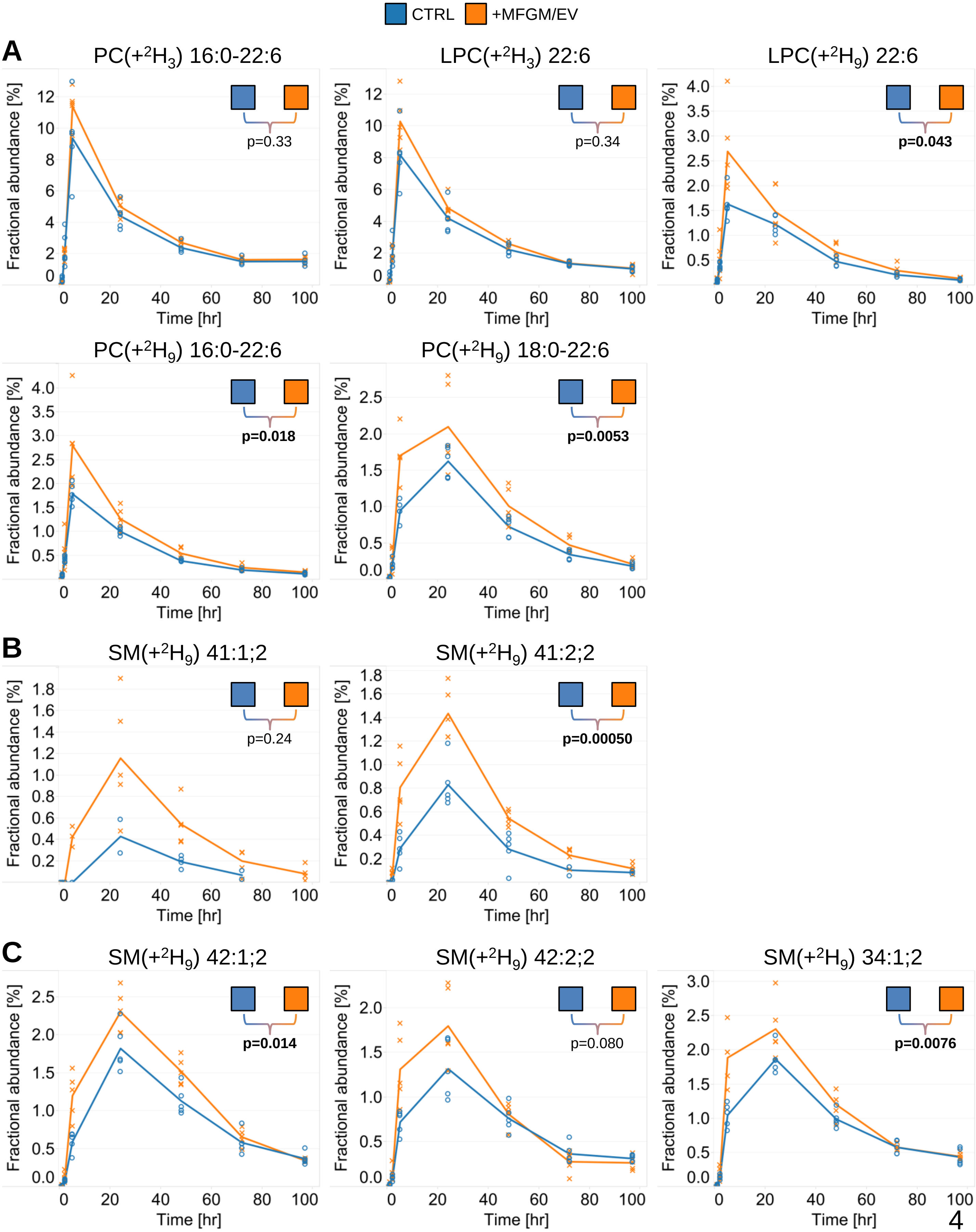
CTRL
+MFGM/EV
A
PC(+$^2H_3$) 16:0-22:6
p=0.33
LPC(+$^2H_3$) 22:6
p=0.34
LPC(+$^2H_9$) 22:6
p=0.043
PC(+$^2H_9$) 16:0-22:6
p=0.018
PC(+$^2H_9$) 18:0-22:6
p=0.0053
B
SM(+$^2H_9$) 41:1;2
p=0.24
SM(+$^2H_9$) 41:2;2
p=0.00050
C
SM(+$^2H_9$) 42:1;2
p=0.014
SM(+$^2H_9$) 42:2;2
p=0.080
SM(+$^2H_9$) 34:1;2
p=0.0076
Fractional abundance [%]
Time [hr]

Figure S5. The MFGM/EV ingredient increases lipid metabolic activity. Related to Figure 7.

A-C) Representative timelines for indicated PC and SM molecules. Lines represent means and crosses and circles show individual values (n = 2-5 rats/timepoint/dietary group). Statistical analysis was done by repeated measures ANOVA F-test. Significant timelines are highlighted in bold (p-value < 0.05).
