# Supplementary material for: Dietary Intake of a Milk Sphingolipid-Rich MFGM/EV Concentrate Ameliorates Age-Related Metabolic Dysfunction
Source: Nutrients. 2025 Jul 31;17(15):2529. doi: 10.3390/nu17152529 (PMC12348648; doi:10.3390/nu17152529)
Supplement: Supplementary file 1 [file nutrients-17-02529-s001.zip › Table S3_MRM transitions.pdf]

Table S3. Multiple reaction monitoring (MRM) parameters used for proteotypic peptide detection.

Protein	Peptide sequence	MRM (*m/z*)	Cone/Collision (V/eV)
ApoB48	LSQLETYA	519.6 → 467.1 (132.1, 203.1)	30 / 10
ApoB48 (IS)	LSQLETYA-[$^{13}C_6{}^{15}N$]K	523.1 → 474.1 (139.1, 203.1)	30 / 10
ApoB100	ASEAVYDYVK	633.4 → 471.1 (681.3)	30 / 18
ApoB100 (IS)	ASEAVYDYV-[$^{13}C_6{}^{15}N_2$]K	637.4 → 479.1 (689.3)	30 / 18

IS, internal standard. Parentheses indicate the fragment ions used as qualifiers.
